# Detecting *de Novo* Homoeologous Recombination Events in Cultivated *Brassica napus* Using a Genome-Wide SNP Array

**DOI:** 10.1534/g3.118.200118

**Published:** 2018-06-15

**Authors:** Erin E. Higgins, Wayne E. Clarke, Elaine C. Howell, Susan J. Armstrong, Isobel A. P. Parkin

**Affiliations:** * Agriculture and Agri-Food Canada, 107 Science Place, Saskatoon, SK, S7N 0X2, Canada; † School of Biosciences, University of Birmingham, Edgbaston, Birmingham B15 2TT, UK

**Keywords:** recombination, chromosomal rearrangement, *Brassica napus*, neopolyploid, duplication/deletion

## Abstract

The heavy selection pressure due to intensive breeding of *Brassica napus* has created a narrow gene pool, limiting the ability to produce improved varieties through crosses between *B. napus* cultivars. One mechanism that has contributed to the adaptation of important agronomic traits in the allotetraploid *B. napus* has been chromosomal rearrangements resulting from homoeologous recombination between the constituent A and C diploid genomes. Determining the rate and distribution of such events in natural *B. napus* will assist efforts to understand and potentially manipulate this phenomenon. The Brassica high-density 60K SNP array, which provides genome-wide coverage for assessment of recombination events, was used to assay 254 individuals derived from 11 diverse cultivated spring type *B. napus*. These analyses identified reciprocal allele gain and loss between the A and C genomes and allowed visualization of *de novo* homoeologous recombination events across the *B. napus* genome. The events ranged from loss/gain of 0.09 Mb to entire chromosomes, with almost 5% aneuploidy observed across all gametes. There was a bias toward sub-telomeric exchanges leading to genome homogenization at chromosome termini. The A genome replaced the C genome in 66% of events, and also featured more dominantly in gain of whole chromosomes. These analyses indicate *de novo* homoeologous recombination is a continuous source of variation in established *Brassica napus* and the rate of observed events appears to vary with genetic background. The Brassica 60K SNP array will be a useful tool in further study and manipulation of this phenomenon.

The genomic relationship between the major Brassica species was first described by U. N. (1935) and is defined by three diploid species: *B. rapa* (A genome), *B. nigra* (B genome) and *B. oleracea* (C genome); and three allotetraploids created from each pair-wise hybridization of these genomes: *B. juncea* (A and B genomes), *B. napus* (A and C genomes) and *B. carinata* (B and C genomes). Of these Brassica species, *B. napus* (canola or oilseed rape) is the most economically important and is believed to have been formed in the last 10,000 years centered around Mediterranean Europe (Chalhoub *et al.* 2014) and is now grown on all continents, harvested predominantly for its oil. The Brassica species provide an excellent platform for the study of genome evolution in polyploids since they encompass multiple ancient genome duplication events (Chalhoub *et al.* 2014). These events include the gamma triplication event common to most eudicots, the α and β whole genome duplication common to all Brassicaceae, a Brassica lineage specific whole genome triplication that led to the formation of the Brassica diploids (or mesopolyploids), and most recently whole genome hybridization resulting in the three allopolyploid (or neopolyploid) species (Masterson 1994; Bowers *et al.* 2003; Lysak *et al.* 2005; Schranz *et al.* 2006).

Polyploid formation leads to a phase of genomic shock in response to the duplication of all genes, with concomitant gene balance and regulatory issues (Wendel 2000; Doyle *et al.* 2008). During meiosis, homologous chromosomes pair and the ensuing recombination facilitates the production of viable gametes, each with a complete set of chromosomes. However, in neopolyploids formed from closely related species, a chromosome may have more than one potential pairing partner, termed a homoeologue. Cytological analysis of meiotic cells in neopolyploids shows formation of bivalents, unpaired univalents and multivalents of homologous and homoeologous chromosomes (Attia and Röbbelen 1986b). Recombination between homoeologous chromosomes where all or part of a chromosome from one genome is replaced with the homologous regions from the second genome can result in inheritance of either the recombined segments from both homoeologues, with no apparent loss of genetic material, or only one of the recombined segments, leading to gain and loss of genetic material. The latter events have often been termed homoeologous non-reciprocal translocations (HNRT), although by their nature they are derived from reciprocal exchange, to prevent confusion such events will be referred to as homoeologous recombination (HeR) events or exchanges. Multivalent and univalent formation can also lead to unbalanced gametes. During anaphase I unpaired chromosomes either move to one pole or are split between the poles by the spindle apparatus. The complicated dissolution of multivalents leads to unpredictable separation of chromosomes, most of which will result in unbalanced gametes (Zamariola *et al.* 2014). Such gametes can be nonviable or the resultant embryos may produce plants that are sterile or unfit for their current environment resulting in an overall decrease in yield (Jenczewski and Alix 2004). The mechanisms responsible for the genetic stabilization (or diploidisation) of neopolyploids are of interest for maintaining fitness in crops, limiting gene flow to native plants, and exploiting the diploid gene pools of polyploid progenitors for novel traits.

Polyploidy is very common in plants, including several important crop species such as wheat, cotton, canola, coffee and peanut, and evidence exists for at least some level of genetic control of chromosome pairing in all of these species (Cifuentes *et al.* 2010; Lashermes *et al.* 2016; Nguepjop *et al.* 2016). The most well characterized of these is wheat where chromosome pairing control has been studied since the 1950s after discovery of the *Pairing homoeologous1 (Ph1)* locus that had a major effect on the control of homoeologue pairing and recombination (Riley and Chapman 1958). Using cytology it was observed that in plants lacking the *Ph1* locus there were more univalents and multivalents at metaphase I of meiotic cells rather than the typical prevalence of homologous bivalents, but a precise mechanism for this phenotype continues to be investigated (see (Greer *et al.* 2012; Bhullar *et al.* 2014; Martín *et al.* 2017; Rey *et al.* 2017) for recent work). Though fewer studies have focused on the genetic control of pairing in Brassica, it is an excellent system for studying pairing control and homoeologous recombination because the allotetraploid species of the triangle of U (U. N. 1935) can be recreated through crossing and subsequent chromosome doubling of the two constituent diploid species (Snowdon 2007). Researchers have successfully used sequential fluorescence *in situ* hybridization (FISH) and genomic *in situ* hybridization (GISH) to distinguish the A and C genome chromosomes in *B. napus* (Howell *et al.* 2008), and a novel chromosome painting technique was used to identify all of the chromosomes from *B. rapa*, *B. napus* and *B. oleracea* (Xiong and Pires 2011). This makes it possible to not only identify homoeologous bivalents and multivalents but to pinpoint the chromosomes preferentially pairing in meiotic cells. Cytological analysis previously identified a major quantitative trait locus (QTL) that contributed to variation in homoeologous chromosome pairing in allohaploid *B. napus* plants of two genotypes (Jenczewski *et al.* 2003). In addition, this locus appeared to impact homologous recombination; however, it did not seem to contribute to variable homoeologous pairing in allotetraploids (diploids) of the same *B. napus* lines (Nicolas *et al.* 2009).

Molecular markers have previously been used to identify homoeologous recombination events in *B. napus* (Parkin *et al.* 1995; Sharpe *et al.* 1995; Udall *et al.* 2005; Rousseau-Gueutin *et al.* 2017; Stein *et al.* 2017). By visualizing both A and C genome loci with restriction fragment length polymorphism (RFLP) markers it was possible to resolve homoeologous recombination events by the gain of an allele at one locus coupled with loss of an allele at the homoeologous locus (Parkin *et al.* 1995). This simultaneous gain and loss of alleles at genetically linked loci on homoeologous chromosomes provided evidence of HeR events. Such analyses of a population derived from a cross between a newly resynthesized *B. napus* (created by crossing a *B. rapa* and *B. oleracea* line followed by chromosome doubling to produce an allotetraploid) and an established *B. napus* parent line showed a significant increase in homoeologous recombination between the A and C genomes relative to a population derived from a cross between two adapted *B. napus* parent lines (Parkin *et al.* 1995; Sharpe *et al.* 1995). While highly reproducible the laborious nature of RFLP markers makes them difficult to assess for a large number of lines across the whole genome. Simple sequence repeat (SSR) markers have been used to show reciprocal gain and loss of A1 and C1 loci in progeny of a resynthesised *B. napus* (Szadkowski *et al.* 2010) but SSRs offer only a marginal advantage in assay time compared to RFLP markers. The development of the Brassica 60K Infinium single nucleotide polymorphism (SNP) array (Clarke *et al.* 2016) provides a high-density genome-wide platform to assess homoeologous recombination. SNP arrays allow genotyping of hundreds of lines at thousands of loci in a matter of days and have been successfully used for genetic mapping in bi-parental populations (Liu *et al.* 2013; Wang *et al.* 2015; Yang *et al.* 2017), differentiating between the different Brassica species of U’s triangle (Mason *et al.* 2015), identification of parental alleles in interspecific Brassica species (Mason *et al.* 2014), and genome wide association studies (GWAS) on diverse sets of *B. napus* germplasm (Hatzig *et al.* 2015; Körber *et al.* 2015). Use of the Brassica 60K array to identify segmental deletions in resynthesized *B. napus* has been combined with cytological analysis to identify translocations caused by homoeologous recombination in resynthesized *B. napus* individuals (Rousseau-Gueutin *et al.* 2017). Similarly, the lack of amplification at physically linked SNP loci was used in conjunction with re-sequencing data to reveal homoeologous exchanges underlying QTL for *B. napus* seed quality traits (Stein *et al.* 2017).

This paper describes use of the Brassica 60K SNP array to identify *de novo* homoeologous recombination events in allotetraploid *B. napus*. The high-density coverage provided by the array allows for genome-wide detection of recombination events at a greater depth and higher resolution than previous marker-based assays. The efficacy of this method was tested by assaying levels of *de novo* homoeologous recombination in 10 testcross populations derived from established *B. napus* lines. These data provide a range of expected levels for such events in *B. napus* and define genomic regions more prone to homoeologous recombination.

## Materials and Methods

### Testcross population development

Ten *B. napus* lines were chosen from a collection of spring-type cultivars (ACSRsyn1, Bronowski, Daichousen (fuku), Maris Haplona, PAK85912, Surpass 400, Svalof’s Gulle, Topas, Tribune, Zhongyou 821) based on diverse geographical distribution (Canada, Poland, Korea, United Kingdom, Pakistan, Australia, Sweden, Canada, Australia, China, respectively) and where available molecular information (Bus *et al.* 2011). Formation of ACSRsyn1 was created by crossing a *B. napus*/*B. oleracea* triploid with a *B. napus/B. rapa* triploid (DTN-1/*B. alboglabra* 89-5402//DTN-1/*B. rapa* Parkland) and selecting for an individual with a complete AACC genome followed by selfing for several generations (provided by Sally Vail, Agriculture and Agri-Food Canada, Saskatoon). Plants were grown in a greenhouse at 18° with a 16/8 hr photoperiod (day/night). Hand pollinations were used to cross the *B. napus* individuals with the Australian *B. napus* cultivar “Rainbow” to produce a testcross population for each line. Young leaf tissue of sixteen individuals for each of the populations was harvested and freeze-dried for DNA extraction. Three of the testcross populations, PAK85912, Zhongyou821 and Maris Haplona were expanded to 48 individuals each, though two of the PAK85912 progeny were determined to be selfs and were disregarded from the data set.

### Brassica SNP array

High quality DNA was extracted from freeze-dried leaf tissue using a cetyltrimethylammonium bromide (CTAB) based method (Murray and Thompson 1980). DNA was quantified with the Quant-it Picogreen dsDNA assay kit (Life Technologies Inc., Burlington ON, Canada) and 200 ng was hybridized to the Brassica 60K Infinium array (Clarke *et al.* 2016) as described in the manufacturer’s protocol (Illumina Inc., San Diego, CA). The arrays were scanned using an Illumina HiScan and SNP data were analyzed using the genotyping module of the GenomeStudio software package (Illumina Inc.) using default settings with the exception of the no-call threshold, which was set at 0.05 and a custom cluster file was applied (Clarke *et al.* 2016). The software creates a two-dimensional image for each SNP marker where the graphical position of each individual is determined by the fluorescent intensity (R value - y axis) and the ratio of the two allele-specific fluorophores (θ value – x axis). Individuals are assigned a genotype based on their position in the graph. The software is designed for diploid species with two alleles at each locus (AA/BB) so in a typical cross between two homozygous parents, a classic three cluster profile is produced, the AA and BB clusters would reflect the genotype of each parent and the AB cluster would represent heterozygous progeny. Single copy SNP markers were pre-selected by aligning the flanking sequence provided in the manifest file for the Brassica 60K array to the *B. rapa* and *B. oleracea* genome assemblies (Wang *et al.* 2011; Parkin *et al.* 2014) using BLAT (Kent 2002) and selecting those markers with >90% identity in one diploid genome and <90% identity in the other diploid genome. This resulted in a set of 38,970 markers that were then filtered for polymorphism between the parents of each population. The level of SNP polymorphism for each testcross population is given in Table 1 for each linkage group and the GenomeStudio exported SNP data for each population is provided in Supplementary Tables S1-S10. Three or more consecutive missing or duplicated SNPs were used to identify affected regions; however, the majority of the events (91.5%) were defined by 10 or more physically linked SNP loci (Supplementary Table S11). HeR events were identified by analysis of the homoeologous regions in the testcross individuals as described in the RESULTS.

**Table 1:** Summary of distribution of polymorphic SNP markers per chromosome for each testcross population

Linkage Group	ACSRsyn1	Bronowski	Daichousen (fuku)	Maris Haplona	PAK85912	Surpass 400	Svalof’s Gulle	Topas	Tribune	Zhongyou 821
A1 (21.7 Mb)	631	653	572	715	641	741	537	511	711	560
A2 (29.6 Mb)	380	274	443	441	441	331	344	432	264	455
A3 (35.8 Mb)	875	999	799	761	1012	941	837	849	776	1090
A4 (21.1 Mb)	653	670	565	755	804	655	720	746	623	618
A5 (25.7 Mb)	864	680	552	868	700	552	878	884	371	490
A6 (26.1 Mb)	787	552	665	779	648	790	728	759	692	772
A7 (25.5 Mb)	738	424	647	715	630	771	690	672	684	722
A8 (21.7 Mb)	576	383	280	599	617	605	388	575	523	441
A9 (40.5 Mb)	578	745	865	581	647	535	651	505	495	1051
A10 (17.9 Mb)	359	428	520	360	454	461	481	377	463	533
C1 (45.6 Mb)	1793	1803	1631	1914	820	1008	825	1842	1976	1683
C2 (47.3 Mb)	1145	2685	2087	1239	1857	1312	1162	1504	1316	1807
C3 (67.8 Mb)	1603	2098	1985	1620	1567	2060	1609	1631	1645	1492
C4 (55.1 Mb)	3062	1304	3315	1528	2434	3206	1273	3072	1837	3305
C5 (48.7 Mb)	970	974	572	969	828	627	915	898	706	916
C6 (40.8 Mb)	736	872	828	746	792	747	798	762	800	830
C7 (48.8 Mb)	939	802	1631	991	1204	627	1103	915	1236	1275
C8 (44.7 Mb)	1217	1196	1432	729	1109	942	1042	1120	1309	1379
C9 (56.0 Mb)	757	780	1126	786	753	730	872	690	766	966
**TOTAL**	18663	18322	20515	17096	17958	17641	15853	18744	17193	20385

### Identification of homoeologous regions from the SNP array

The results of the BLAT alignment from aligning the flanking sequences from the SNP loci to the *B. rapa* and *B. oleracea* genome assemblies was also used to identify the top hit in each of the A and C genomes for each SNP probe. Probes with at least 50% identity in both diploid genomes were selected, resulting in 28,334 SNP markers mapped to the A and C genome (Supplementary Table S12) that could be used to determine the homoeologous alignment of the A and C genomes.

### Detection of inherited HeR events using whole genome shotgun (WGS) data

DNA from Zhongyou821 was extracted from nuclei according to Parkin *et al.* (2014). A short insert (350 bp) Illumina DNA sequencing library was constructed according to the manufacturer’s instructions (Illumina, Inc.) and 125 bp paired end (PE) data were generated on the HiSeq2000 platform, providing in total 111 million (M) PE reads (estimated 23x coverage of 1200 Mb genome). Trimmomatic v0.32 (Bolger *et al.* 2014) with the following parameters, LEADING:15 TRAILING:15 SLIDINGWINDOW:4:15 MINLEN:55, was used to remove low quality reads, short inserts and adapter sequences, resulting in 103 M high quality PE reads. A combined pseudo-reference genome was generated from concatenating the *B. rapa* and *B. oleracea* genome assemblies (Wang *et al.* 2011; Parkin *et al.* 2014). Bowtie2 v2.3.3.1 (Langmead and Salzberg 2012) was used to align PE data to the pseudo-reference using the following parameters --local --sensitive --phred33 --minins 0 --maxins 1000 --no-mixed --no-discordant --no-unal --k 20 --dovetail. A custom perl script was used to retain the best alignment for each read as long as the next hit was significantly lower in call stringency. The overall alignment rate was 90.05%, with 92.8 M mapped PE reads. The resultant alignment file was analyzed using the R scripts described in Samans *et al.* (2017), which normalize the read depth across the length of each chromosome, identify regions of the genome where the read depth significantly differs from the chromosomal mean (at 1.5 SD), and finally compare homoeologous regions to define potential fixed HeR events.

### Data availability

Tables S1-S10 contain the SNP marker data for all testcross populations. Table S11 lists all HeR, duplication and deletion events found in the testcross populations. Table S12 provides details of the SNP markers used for homoeologous alignment of A and C genomes. Table S13 shows the compressed SNP data which summarizes *de novo* chromosome gain and loss in each testcross individual. Table S14 lists HeR events in Zhongyou821 identified through whole genome sequencing. The WGS data for Zhongyou821 has been uploaded to the NCBI short read data archive (https://trace.ncbi.nlm.nih.gov/) under BioProject ID PRJNA454160. Supplemental material available at Figshare: https://doi.org/10.25387/g3.6207530.

## Results

### Dissecting SNP marker patterns to identify homoeologous recombination events

Recombination rates for an individual can be determined by studying the products of meiosis or the genotypes of resulting progeny in the subsequent generation. Ten test populations were derived by crossing ten spring-type *B. napus* lines with the Australian *B. napus* cultivar Rainbow. In total, 256 individuals were assayed with the Brassica 60K SNP array, two lines were identified as self progeny. Initially 16 individuals for each of the 10 *B. napus* testcross populations were assessed and three populations with differing levels of observed events, PAK85912, Maris Haplona and Zhongyou821, were expanded to 46-48 individuals each. For each individual, the meiosis of both the *B. napus* line and the testcross parent Rainbow could be assessed, meaning the products of 508 meioses were evaluated.

Although the nature of polyploid genomes can impact the use of hybridization based tools such as Infinium arrays, the Brassica 60K SNP array was designed such that ∼58% of the loci on the array were estimated to amplify a single genome (Clarke *et al.* 2016). However, conversely as much as 32% of the SNPs on the array identify both an A and C genome locus. Although intuitively these loci might appear useful in the analyses of homoeologous recombination, as detailed in Mason *et al.* (2017) since both loci use the same two fluorophores for the A and B alleles, they cannot readily be distinguished, thus such loci were eliminated from the analyses.

**Figure 1:**
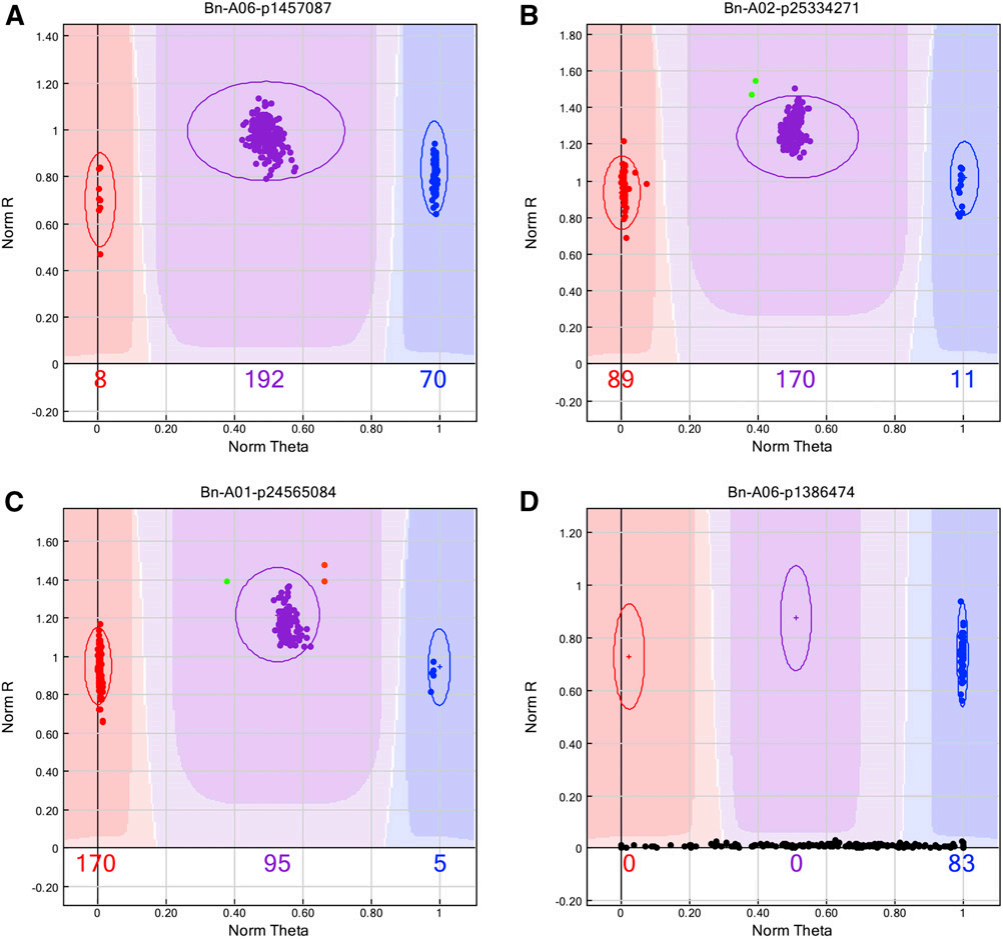
Range of observed SNP patterns in *Brassica napus* testcross individuals. A) GenomeStudio image of a typical three cluster SNP with Parent 1 in red (genotype AA), Parent 2 in blue (genotype BB) and all testcross individuals of polymorphic populations in purple (genotype AB); B) GenomeStudio image of four cluster SNP with two AAB individuals shown in green; C) GenomeStudio image of five cluster SNP with one AAB individual shown in green and two ABB individuals shown in orange; D) GenomeStudio image of a SNP with no amplification in Parent 1 therefore all testcross individuals in polymorphic crosses have genotype B0 and are in the BB parental cluster.

Since homoeologous recombination events are relatively rare, affecting only a small part of the genome, the most common pattern observed for single locus assays, approximately 90% of the time, in all populations was the typical three cluster pattern, AA/AB/BB, expected for an F_1_ with no gain or loss of alleles in the testcross individuals. The two parents were found in the AA and BB clusters respectively and all the progeny were found in the heterozygote cluster, suggesting normal homologous recombination and segregation had occurred during meiosis in the testcross parents (Figure 1A).

The gain and loss of alleles due to homoeologous recombination created more complicated cluster patterns, polymorphic SNPs expected to amplify a single genome (A or C), produced patterns with the expected three, but also one, four and five distinct clusters and were used to score the testcross populations. In each population, between 4–19% of the polymorphic markers showed these aberrant cluster patterns. These loci had the hallmarks of single copy SNPs, with parental alleles optimally separated (AA θ value <0.15, BB θ value >0.85) and heterozygote genotypes falling equi-distant between the two, yet additional clusters were observed across the horizontal plane. Those showing four distinct clusters had testcross individuals falling into three different groups: 1) with either one of the two parents; 2) in the expected AB cluster; or c) in a new cluster between the AB group and one of the parents (Figure 1B). These patterns can be explained by the gain or loss of alleles due to homoeologous recombination in one of the parents. Segregation of testcross individuals with either parent indicates they are missing an allele that should have been inherited from the other parent. Since these lines only carry an allele from one parent they were designated as genotype A0 (or B0). Individuals in the expected heterozygote cluster are presumed to have inherited one allele from each parent, and are therefore genotype AB. Based on the position of the new cluster between one of the parents and the AB group, those individuals are presumed to have inherited one allele from the first parent and two copies of the allele from the second parent and are therefore genotype ABB (or AAB). In other cases individuals were observed on both sides of the heterozygote cluster resulting in five clusters on the SNP image, indicating there were homoeologous exchanges occurring in both Rainbow and the other *B. napus* parent, which were inherited in some of the individuals, so all five genotypes, A0, AAB, AB, ABB and B0 are represented (Figure 1C). Lastly, some SNPs had a single cluster containing one parent and all of the testcross individuals while the other parent showed no amplification, indicating the absence of the SNP locus in one of the parents (Figure 1D).

### Defining the genomic position of the homoeologous recombination (HeR) events

**Figure 2:**
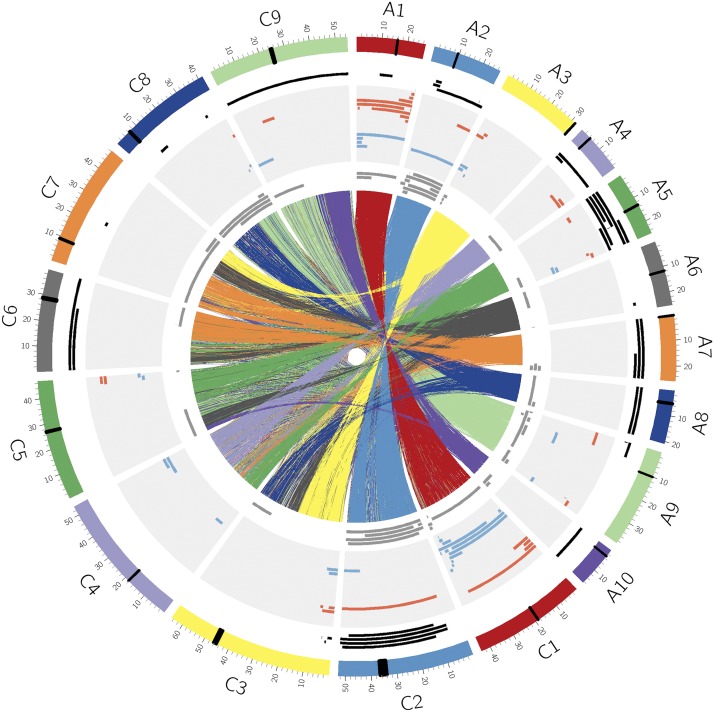
Circos plot (Krzywinski *et al.* 2009) depicting alignment of the *B. rapa* and *B. oleracea* genomes, and summary of *de novo* homoeologous recombination events in 11 *B. napus* genotypes. The *B. rapa* chromosomes A1-A10 and *B. oleracea* chromosomes C1-C9 are shown in the outer ring. Homoeologous regions between the chromosomes as identified by the sequence homology of SNP probes are shown as colored links drawn in the center of the image. The three rings internal to the chromosomes show from outer to inner: duplication events as black tiles; chromosomal gain and loss due to *de novo* HeR as red and blue tiles, respectively; and deletions as gray tiles. The vertical black line on each chromosome represents the approximate centromere position (Wang *et al.* 2011; Parkin *et al.* 2014).

Due to the genome specificity of the SNP assays and filtering of the SNP loci, the A and C genome loci were assessed independently, thus the gain/loss of homoeologous loci was not captured simultaneously by any one SNP assay. Homoeologous recombination (HeR) events by definition result in the exchange of chromosomal material between syntenic regions of the A and C genome within *B. napus*. Because only a single A or C genome locus can be scored for each SNP, it was necessary to first identify the syntenic regions between the genomes that can be identified by the SNP loci on the array, and then look for reciprocal allele gain and loss at SNP loci in those regions, thus determining the level and distribution of HeR events. The flanking sequences of the SNPs on the Brassica 60K array were aligned against the genomic sequence of *B. rapa* (A genome) and *B. oleracea* (C genome) which produced a clear alignment of homoeologues with comprehensive genome coverage that was in concordance with previous analyses (Parkin *et al.* 2014) (Figure 2). In some cases two chromosomes were entirely aligned along their length, for example A1 and C1, while others had two or more homoeologous partners, such as A9 and A10, which were aligned with the top and bottom of C9, respectively.

Genome-wide coverage and density of SNP markers along the chromosomes is important to ensure there is sufficient polymorphism between Rainbow and the 10 *B. napus* testcross parents because allele gain and loss can only be visualized when the two parents have opposing alleles. The large number of usable markers on the Brassica 60K array maximized the ability to identify homoeologous regions and determine if an event involved the two homoeologues or if only one genome was duplicated or deleted. The number of informative polymorphic markers in each testcross population is summarized in Table 1 and complete genotype information for all SNP markers is shown in Supplementary Tables S1-S10.

For each of the testcross individuals the genotypes for each A genome SNP locus were aligned with the genotypes for the SNPs from the homoeologous region of the C genome to allow ready visualization of the reciprocal allele gain and loss. For a given individual, when the SNP loci from say the C genome showed an extra copy of the C genome allele from one parent (scored as ABB or AAB), the allele from the corresponding region in the A genome was missing (scored as A0 or B0) (Supplementary Table S13). This gain/loss pattern was observed along both homoeologues for at least three physically linked SNP markers in order to confidently determine a HeR exchange had occurred. The number of physically linked loci identifying HeR affected regions ranged from 3-1879, with only four events defined by the minimum number of loci, and the majority of the rearrangements (91%; 151/165 affected regions) were identified by 10 or more physically linked loci (Supplementary Table S11).

### Confirmation of fixed HeR events detected with the SNP array

**Figure 3:**
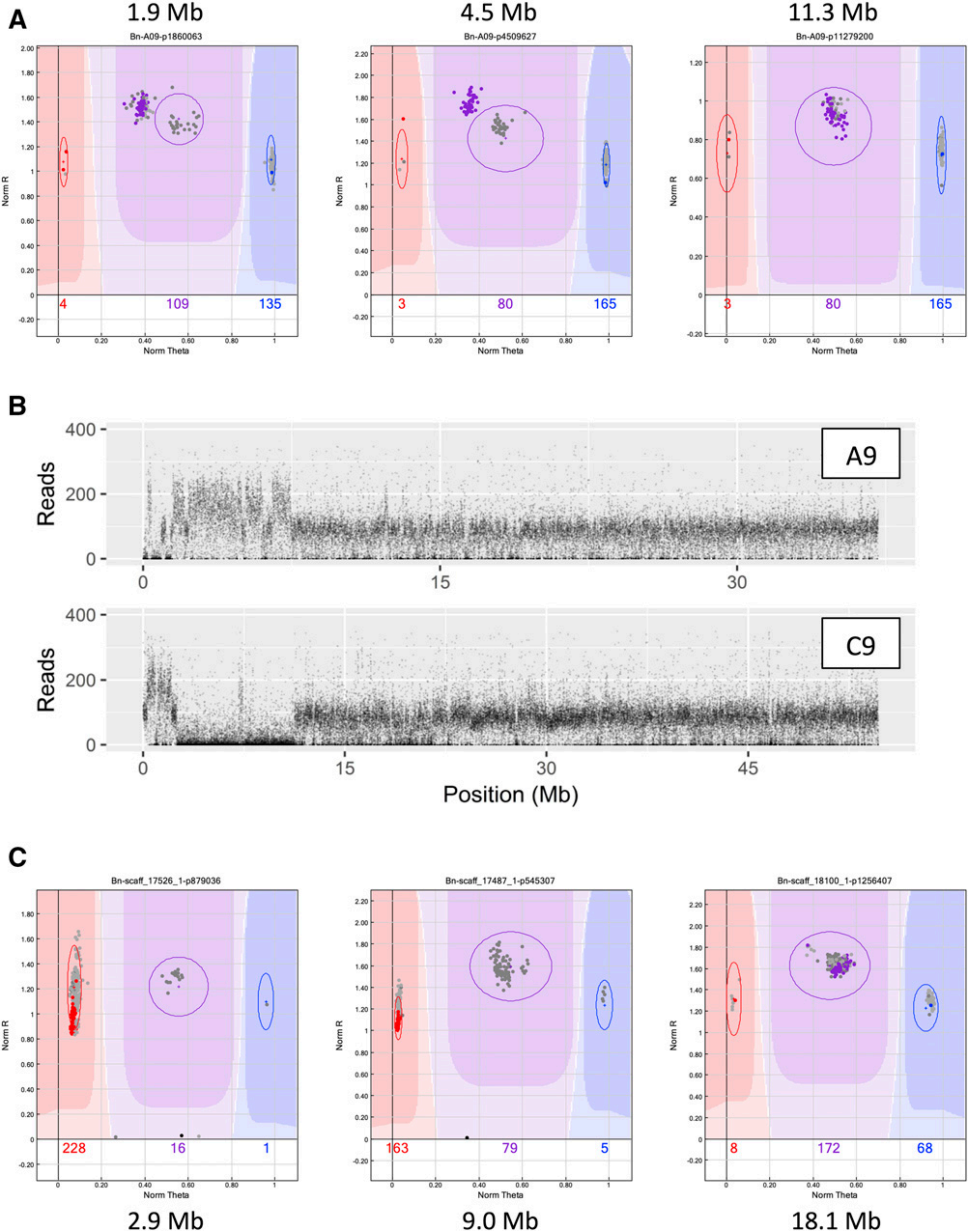
Identification of a fixed HeR event in Zhongyou821 using SNP array data and confirmed through whole genome re-sequencing. A) GenomeStudio images from A9; the SNP loci sampled at 1.9 Mb and 4.5 Mb show all testcross individuals (purple) in the AAB cluster biased toward the Zhongyou821 parent (red) and away from Rainbow (blue). Individuals in gray in the expected AB cluster are from other test cross populations without the genome rearrangement. Below the rearrangement (example at 11.3 Mb) normal SNP patterns were observed. B) Plot of normalized sequence read depth along the A9/C9 chromosomes from whole genome shotgun sequencing data of Zhongyou821. On A9 the first Mb is deleted and 2-8 Mb is duplicated, as opposed to C9 where the first Mb is duplicated and 2-11 Mb is deleted. C) GenomeStudio images from C9 showing no amplification of loci in Zhongyou821 (black) at 2.9 Mb and 9.0 Mb, while all testcross individuals (red) cluster with the Rainbow parent. At 18.1 Mb the parents are in the AA and BB clusters and the testcross lines (purple) are AB. The lone individual to the left is Zhongyou821 testcross line #26, which has an extra C9 chromosome and therefore still has the genotype AAB.

Recent studies using whole genome (Chalhoub *et al.* 2014; Samans *et al.* 2017) and transcriptome sequencing (He *et al.* 2016; Lloyd *et al.* 2017) show evidence of historical HeR events which have become fixed in modern *B. napus* varieties. These types of events could be seen in the current analyses, where all individuals of a testcross family are not in the expected central heterozygous cluster for a set of homoeologous SNP loci, but are biased toward one parent at SNP loci for one genome while at the homoeologous loci, all individuals are in the other parental homozygous cluster, indicating a duplication and deletion, respectively. Detection of such an event is shown in Figure 3 for a fixed HeR exchange between the A9/C9 chromosomes in the Zhongyou821 population. Whole genome shotgun sequencing of Zhongyou821 verified the presence of this event, as evidenced by a significant increase and decrease in normalized read depth as determined through sequence alignment to the A9 and C9 chromosomes, respectively (Figure 3). Of the eight such HeR events that could be resolved using the sequence analyses, five were detected in the SNP array data, ranging in size from one to nine Mb (Supplementary Table S14). These data provided confirmatory evidence that SNP array analysis of F_1_ populations can be used to identify historical HeR fixed events in *B. napus* lines.

### De novo homoeologous recombination in B. napus

The focus of the study was to identify *de novo* rearrangements and all further discussion and calculations are based on new HeR exchanges. Such events are seen only in a single individual in a given testcross population and are presumed to have happened during the meiosis that produced the F_1_ gamete. Similarly in cases where the same duplication or deletion event was seen in more than one individual within a population the event was assumed to have been present but not yet fixed (or functionally heterozygous) in the parental line and thus only inherited in a subset of the population and was not considered in subsequent analysis. Generally these events seemed to occur at similar levels to *de novo* events and testcross populations with high levels of *de novo* HeR also exhibited high levels of these segregating events (Tables S1-S10).

The products of 508 meioses were examined and 36 *de novo* events were found where both the deletion and duplication of SNP loci in the corresponding homoeologous regions could be detected (Figure 2, Table 2). In 54 instances only deletion of linked loci was observed and similarly in 39 cases only duplicated linked loci were evident (Figure 2, Table 2). These unpaired duplications and deletions were not unexpected since cytological observations of pairing control in newly resynthesized *B. napus* has frequently shown bivalents, multivalents and unpaired univalent chromosomes (Attia and Röbbelen 1986b; Szadkowski *et al.* 2010; Rousseau-Gueutin *et al.* 2017), so it was expected there could be allele gain and loss due to the complicated dissolution of multivalents, and the segregation of unpaired chromosomes. Although such events indicate aberrant pairing only the 28% of observed events with the reciprocal gain and loss of multiple physically linked loci could be reliably attributed to homoeologous recombination in the testcross parent(s). Of the 93 events for which only gain or loss was visible in the marker data, 27% could be attributed to aneuploid individuals with an additional (13) or missing (12) chromosome (Table 2). As identified in previous studies (Rousseau-Gueutin *et al.* 2017; Stein *et al.* 2017) deletion events are easily visible in the genotype data output from GenomeStudio, but duplication events less so. The current assay identifies individual testcross lines with a duplication based on their θ value separation from the rest of the population caused by a difference in relative fluorescence of the two SNP alleles (Figure 1B and C). However, since the software can only call AA/AB/BB genotypes these individuals are either automatically called as AB or as a missing value. In the genotype output, multiple linked markers with mismatches and missing calls can indicate a potential duplication, but this must be validated by studying the individual SNP images making it more difficult to identify duplications, particularly very small ones. The smallest HeR event observed was 0.09 Mb, the smallest deletion was 0.04 Mb and the smallest duplication was 0.28 Mb (Table S11). The largest HeR was 40.6 Mb, the largest deletion was 28.6 Mb, and the largest duplication was 35.3 Mb. The size and position of all *de novo* HeR events, deletions and duplications observed in the testcross populations is summarized in Supplementary Table S11 and visualized in Figure 2.

**Table 2:** Summary of recombination events in *B. napus* testcross populations

Line	Number of Individuals	Duplication	Deletion	HeR	Aneuploid	TOTAL
**ACSRsyn1**	16	2	2	0	0	4
**Bronowski**	16	0	1	0	0	1
**Daichousen (fuku)**	16	0	0	1	1	2
**Maris Haplona**	48	1	8	0	1	10
**Surpass 400**	16	0	0	0	0	0
**Svalof’s Gulle**	16	1	0	0	1	2
**Tribune**	16	2	2	0	1	5
**Topas**	16	0	1	0	1	2
**PAK85912**	46	5	4	5	3	17
**Zhongyou821**	48	3	2	0	4	9
**Rainbow**	254	12	22	30	13	77

As expected chromosome exchanges at the ends of chromosomes that require only one recombination (61 events) were more common than internal exchanges that require a second recombination (43 events). Many of the HeR events (36%) as seen for A3/C3, A4/C4, A5/C5, and A9/C8 were effectively terminal, involving gain/loss of the chromosome ends (Figure 2). It was also noted that 12 events spanned the centromere position (excluding aneuploids) and 25 events appeared to have breakpoints localized to the centromere position (Figure 2). Though centromeric breakpoints are not likely due to meiotic recombination, they are presumably a consequence of pairing between homoeologues and subsequent breakage during anaphase I and are therefore still indicative of meiotic abnormalities. In total, including aneuploids, A genome chromosomes or regions were gained 50 times and lost 44 times, while the C genome was duplicated 25 times and lost 46 times. This discrepancy is largely due to the fact that in 66% of the HeR events the A genome chromosome was duplicated and the C genome homoeologue was lost (Table 3).

**Table 3:** Summary of recombination events on each chromosome from the 10 *B. napus* testcross populations

Chromosome	Duplication	Deletion	HeR Gain	HeR Loss	Aneuploid	TOTAL
A1	3	1	13	4	1	22
A2	3	6	1	1	4	15
A3	0	3	2	3	0	8
A4	1	1	2	0	1	5
A5	6	1	3	2	2	14
A6	1	2	0	0	0	3
A7	1	3	0	0	2	6
A8	0	1	0	0	3	4
A9	2	7	3	2	0	14
A10	0	2	0	0	1	3
C1	0	3	4	13	1	21
C2	2	2	1	1	5	11
C3	3	1	3	2	0	9
C4	0	0	0	3	0	3
C5	0	2	2	2	0	6
C6	1	2	0	0	1	4
C7	1	2	0	0	1	4
C8	2	2	1	2	2	9
C9	0	1	1	1	1	4
TOTAL	26	42	36	36	25	165

Seventeen of the HeR events with concomitant gain and loss of genetic material were between A1 and C1 (47%) though unpaired deletion and duplication events for A1 and C1 were not disproportional (5% and 4%, respectively) compared to other chromosomes (Table 3). The other 53% of HeR events were not distributed evenly across the chromosomes. A9 was involved in five events, three with C8 and two with C9, and though C9 did not recombine with its other potential pairing partner A10, this association was previously observed in allohaploid *B. napus* so pairing of these chromosomes is certainly possible (Grandont *et al.* 2014). All chromosomes had at least one event, though C4 had no unpaired duplications or deletions but had three gain/loss HeR events and A6, A7, A8, A10, C6 and C7 had no gain/loss HeR. Curiously one Maris Haplona individual (#19) had inherited an extra copy of both A7 and C6 from Rainbow. Similar to the bias observed for the HeR events, missing or extra copies of A1/C1 and A2/C2 represented almost half (11/25) of the 25 aneuploid lines, for the reminder of the genome there were more, 9 compared to 5 aneuploid lines involving the A genome, of which 3 lines had an extra A8 chromosome.

Though the *B. napus* lines used are established breeding lines they showed varying rates of *de novo* homoeologous recombinaton. Rainbow and PAK85912 had the highest rates with 22% of individuals having at least one deletion, duplication or gain/loss HeR and Surpass 400 had the lowest rate with no events in the 16 individuals tested. The ACSRsyn1 line was produced through interspecific crossing (see materials and methods for pedigree information) and was expected to have a high rate of homoeologous recombination, but only four events were observed in the 16 testcross lines, which is equivalent to the rates observed in Rainbow and PAK85912 and presumably results from stability selected over multiple generations.

One of the advantages of using the SNP array to detect HeR events is the relatively dense marker coverage as compared to RFLP or SSR markers. Smaller exchanges can be identified and the marker density allows for more precise physical positioning of the breakpoints. Calculating the distance from the identified recombination event to the nearest segregating marker gives an indication of how the density of the markers is an improvement over older marker technologies. In this data set the smallest interval to which the recombination could be positioned was estimated to be 172 bp, while the largest was 8.7 Mb with an average size of 0.4 Mb (Table S11).

## Discussion

Though *B. napus* is an excellent system for studying homoeologous recombination, relatively few studies have analyzed the prevalence of *de novo* events, in part because of the limits of molecular marker technology. However, the high-density array formats for SNP markers make them ideal for quantifying homologous and homoeologous recombination rates, and the depth of coverage of markers on the array helps to overcome the limitations of studying each genome independently.

In the current study, *de novo* HeR was assayed in 508 meioses derived from 11 established *B. napus* lines, of these PAK85912 and Rainbow proved the most unstable. For each line, between 21–22% of the testcross individuals showed evidence of *de novo* segmental chromosome duplication or loss (excluding aneuploidy) presumed to be the result of homoeologous recombination or homoeologous associations. Previous studies of AC amphihaploids have suggested genotype contributes to the observed rates of HeR in *Brassica* species (Attia and Röbbelen 1986a; Attia and Röbbelen 1986b; Jenczewski *et al.* 2003). The study by Udall *et al.* (2005) examined three populations from natural *B. napus* crosses and found the rate of homoeologous recombination in one population was more than twice that of the other two (1.09%, 0.49% and 0.43% of all recombination events were homoeologous). In the 46 PAK85912 testcross lines there were five confirmed HeR events as well as five deletions and 4 duplications that are likely caused by chromosome mispairing, recombination and segregation. Rainbow had 30 HeR exchanges, 22 deletions and 12 duplications in the 254 individuals studied. In contrast, Zhongyou821 only had five duplication/deletion events with no HeR observed in 48 testcross lines. Considering all populations and the increased marker depth, the observed levels are comparable to those seen in a study using RFLP markers by Sharpe *et al.* (1995), where HeR were identified in four of 174 individuals from a *B. napus* DH population derived from a Canadian spring-type and a European winter line. Further studies will be required to assess the true range in absolute rates of *de novo* HeR in established *B. napus*; however, the cumulative evidence that genotype contributes to variation in HeR in established *B. napus* suggests the evolution of a genetic mechanism(s) to control levels of homoeologous recombination, possibly inherited from the progenitor diploids, and the variation between genotypes implies the quantitative nature of this trait.

As observed in other studies of HeR, including fixed and *de novo* events, the genome of origin and chromosome position contribute significantly to prevalence and directionality of the exchange (Xiong *et al.* 2011; Chalhoub *et al.* 2014; He *et al.* 2016; Rousseau-Gueutin *et al.* 2017; Samans *et al.* 2017). Although all chromosomes showed evidence of *de novo* events, 38.8% of all 129 *de novo* events were contributed by the two pairs of homoeologous chromosomes (A1/C1 and A2/C2), which are syntenic along their entire length, indicating the importance of homology in determining efficiency of chromosome exchange. The gain and loss of sub-telomeric regions of homoeologous chromosomes (A3/C3, A4/C4, A5/C5 and A9/C8) was also more widely prevalent, a phenomenon previously referred to as “homogenisation” (Sharpe *et al.* 1995) and corroborates previous work that showed an increase in events with distance from the centromere (Nicolas *et al.* 2012). However, it was also noted that 25 events were co-positioned with centromere locations (Figure 2). Centromeres tend to form breaks in ancestral karyotype blocks (Parkin *et al.* 2005) suggesting they may act as evolutionary breakpoints and chromosome fusion and fission has almost certainly played a significant role in shaping Brassica genomes (Friebe *et al.* 2005; Schranz *et al.* 2006).

**Figure 4:**
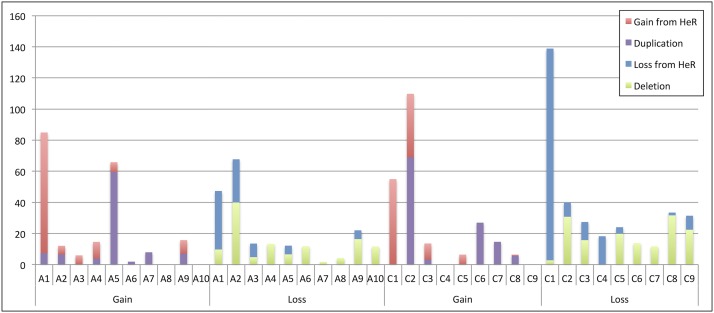
Summary of genomic loss and gain (in Mb) due to deletion, duplication and HeR events for each chromosome. Gain and loss of genetic material due to HeR is shown in red and blue, respectively. Segmental deletions and duplications are shown in green and purple, respectively.

The A genome dominated HeR events such that it replaced the C genome in 66% of events, this bias held with segmental duplications, with 17 of the 26 duplications involving A genome chromosomes. In contrast to Samans *et al.* (2017), *de novo* segmental deletions were found to be higher in the A genome, 27 of the 42 deletions were from the A genome, though the chromosomal material lost was 120 Mb as compared to the C genome, which lost 149 Mb through deletions. This could represent ascertainment bias from the SNP array since proportionally there are a lower number of informative markers per Mb derived from the C genome making it more difficult to detect small deletions (Clarke *et al.* 2016). It was proposed in Samans *et al.* (2017) that since the A genome is ∼25% proportionally smaller than the C genome that the bias toward overall loss of the C genome supports observations of genome size reduction in neo-polyploid evolution, and this was reflected in cumulative amounts of genetic material lost and gained between the sub-genomes (Figure 4). He *et al.* (2016) also evoked the known prevalence of interspecific crossing with *B. rapa* in *B. napus* breeding strategies, which could have led to biased capture of A genome regions by lone C genome chromosomes forming aberrant pairing structures in AAC triploids. One intriguing possibility for the apparent instability of the C genome in *B. napus* is the suggestion that paternal genomes are disproportionally affected in neopolyploids (Lim *et al.* 2007), which would align with suggestions that a relative of *B. rapa* was the possible maternal progenitor of *B. napus* (Allender and King 2010).

Twenty-five aneuploid events were found among the 508 meioses, interestingly the A genome once more featured more dominantly than the C genome, with 14 of the events involving seven of the ten A genome chromosomes, and nine of which resulted in chromosome additions. As with other events A1/C1 and A2/C2 accounted for almost 50% of the events; such aneuploids were prevalent in early generations of resynthesized *B. napus* and chromosome compensation between the highly syntenic chromosomes in the polyploid nucleus is proposed to provide genome balance in the monosomic lines (Xiong *et al.* 2011). Although aneuploids are commonly produced in neopolyploids (Comai 2005) and allopolyploids are known for their genome plasticity (Leitch and Leitch 2008) the level observed (∼5% across all gametes) in established *B. napus* might appear high. However, none of the observed events including the aneuploids are fixed in the testcross lines, and it would be expected that continual selection, for example in the field, for highly fertile euploids would invariably negate the presence of aneuploids and other rearrangements unless they led to a selective advantage. It would be interesting to study the effect of different parental genotypes in the development of hybrids, since it might be expected that high levels of meiotic instability would impact hybrid vigor.

The importance of homoeologous recombination in the developmental history of modern *B. napus* and its establishment as a major oilseed crop became apparent through sequence analyses of the *B. napus* genome that showed a number of important traits for Brassica oil and meal quality were derived from homoeologous recombination events (Chalhoub *et al.* 2014). An indication of continuing genome evolution in *B. napus* was shown when a homoeologous recombination event was found to be segregating in replicate lines from the variety used to generate the *B. napus* genome assembly (Lloyd *et al.* 2017). The current study has shown that these events continue to happen frequently in natural *B. napus*, offering the opportunity to generate novel variation that could be exploited for crop improvement. The use of high-density SNP arrays has become the standard method for genetic mapping in segregating populations, genotyping of elite lines, studies of genotypic diversity, and identification of chromosomal deletions (Liu *et al.* 2013; Hatzig *et al.* 2015; Körber *et al.* 2015; Mason *et al.* 2015; Rousseau-Gueutin *et al.* 2017; Stein *et al.* 2017; Yang *et al.* 2017). The method described in this paper extends the use of SNP array to comprehensively measure the inheritance of homoeologous recombination events. The coverage of the Brassica 60K SNP array makes selection across the whole of the *B. napus* genome possible; in combination with cytological analysis this will present a complete picture of chromosome pairing at meiosis and the resulting homoeologous recombination that affects gamete viability, phenotypic variation and plant fitness. The SNP coverage in concert with the available *B. napus* genome sequence (Chalhoub *et al.* 2014) also allows for a precise identification of recombination hotspots and regions of recombination repression.
